# Loss of heterozygosity at thymidylate synthase locus in Barrett's metaplasia, dysplasia, and carcinoma sequences

**DOI:** 10.1186/1471-2407-9-157

**Published:** 2009-05-21

**Authors:** Hidekazu Kuramochi, Kazumi Uchida, Jeffery H Peters, Daisuke Shimizu, Daniel Vallbohmer, Sylke Schneider, Kathleen D Danenberg, Peter V Danenberg

**Affiliations:** 1 University of Southern California/Norris Comprehensive Cancer Center, 1441 East Lake Ave, Los Angeles, CA, 90033, USA; 2 University of Southern California, Department of Surgery, 1441 East Lake Ave, Los Angeles, CA, USA; 3 Response Genetics Inc, 1640 Marengo Street 620, Los Angeles, CA, 90033, USA; 4 Tokyo Women's Medical University, Institute of Gastroenterology, 8-1 Kawadacho, Shinjuku-ku, Tokyo, Japan; 5 Department of General, Visceral and Cancer Surgery, University of Cologne, Germany

## Abstract

**Background:**

*Thymidylate synthase (TS) *is known to have a unique 28 bp tandemly repeated sequence in the promoter region, and the majorities of subjects have a heterozygous double repeat/triple repeat genotype in their non-cancerous tissue. Loss of heterozygosity (LOH) at the *TS *locus is known to occur in cancer patients, but there is no evidence that it is present in precancerous tissue. The aim of this study was to analyze the frequency and timing of LOH at the *TS *locus in Barrett-associated adenocarcinoma (BA) and its precursory lesions, such as intestinal metaplasia (IM) and dysplasia.

**Methods:**

One hundred twenty-three samples (including 37 with gastroesophageal reflux disease (GERD), 29 with IM, 13 with dysplasia, and 44 with BA) were obtained from 100 patients. Biopsies were obtained from the lower esophageal mucosa/IM/dysplasia/BA, when available. Normal squamous tissue from the upper esophagus was taken as a control. All tissues were analyzed for the *TS *genotype and TS mRNA expression using the real-time reverse-transcription polymerase chain reaction (RT-PCR) method after laser-capture microdissection.

**Results:**

Among the patients with informative heterozygous genotype in their control samples, no sample with LOH at the *TS *locus was observed in the lower esophageal mucosa in GERD patients (0/22 samples). However, 6 out of 21 samples (28.6%) had LOH in IM, 2 of 7 (28.6%) in dysplasia, and 10 of 25 (40.0%) in BA. No significant difference in *TS *mRNA expression levels was observed between *TS *genotypes.

**Conclusion:**

Our results demonstrate that LOH is a relatively frequent and early event in the IM-BA sequence.

## Background

*Thymidylate synthase (TS)*, which is located on 18p11.32, is the only *de novo *source of the thymine base, and its reaction is one of the rate-limiting steps in DNA synthesis [1]. 5-fluorouracil (5-FU) inhibits TS by forming a stable ternary complex among 5,10-methylenetetrahydrofolate, TS, and fluoro-dUMP, the metabolite of 5-FU. Based on this mechanism, the TS expression level is regarded as a predictor of response to 5-FU-based chemotherapy, and patients with low TS reported having better prognoses than those with high TS [2,3]. The *TS *gene is known to have a unique 28-bp tandemly repeated sequence in the 5'-untranslated region (5'-UTR), and is polymorphic in the number of these repetitions [4]. Most individuals have a homozygous double-tandem repeat (2R/2R), a homozygous triple repeat (3R/3R), or a heterozygous (2R/3R) genotype. It has been reported that the 3R/3R genotype is associated with higher levels of *TS *mRNA and/or protein expression than the 2R/2R genotype, suggesting that this 5'-UTR polymorphism can regulate *TS *transcription or translation [5-8]. In addition, a G/C single nucleotide polymorphism (SNP) has been identified within the 3R allele, and segregates the 3R allele into 3RG and 3RC [9,10]. The 3RC allele can abolish the increased transcriptional activity of the 3R variant in vitro by altering a transcription factor-binding site [10]. Recently, another polymorphism, a 6 bp insertion/deletion at bp 1494 in the 3'-untranslated region (3'-UTR) of *TS *gene, was identified [11] and is thought to influence the intratumoral *TS *mRNA level *in vivo *[12].

Using this 5'-UTR polymorphism as a heterozygous marker, we have reported that there is a high incidence of loss of heterozygosity (LOH) at the *TS *locus in colorectal cancer tissue, and have demonstrated that the *TS *genotype in cancer tissue modulated by LOH to give either a 2R/loss or 3R/loss situation influences the chemosensitivity to 5-FU-related drugs and the prognosis of colorectal cancer patients [13]. However, when and how this LOH occurs in cancer cells has never been properly evaluated. If it occurs at an early stage of carcinogenesis, it may be used not only as a predictive marker of chemotherapy response but also as a good biomarker for early cancer detection or for identifying high-cancer-risk patients. Barrett's-associated adenocarcinoma exhibits a good sequence in which to examine this question because the stepwise development of this carcinoma has been well defined.

Barrett's esophagus, which is defined as the replacement of the normal squamous epithelium of the lower esophagus by metaplastic columnar epithelium, occurs in patients with chronic gastroesophageal reflux [14]. The cancer risk of patients with this condition is estimated to be 125 times as high as that of the general population [15], and it predisposes a patient to dysplasia and eventually adenocarcinoma [16]. Surveillance of Barrett's esophagus patients is conducted by frequent and regular endoscopic examinations, but several studies have shown that Barrett's esophagus does not progress to cancer in most patients [17-19]. Thus, most patients will not benefit from endoscopic surveillance because their lesions will not become cancerous during their lifetimes [20]. These observations indicate the need for objective and precise biomarkers of neoplastic progression in Barrett's esophagus.

In this study, frequencies of LOH at *TS *loci were evaluated in each step of carcinogenesis, such as reflux esophagitis, intestinal metaplasia (IM), dysplasia, and Barrett's-associated adenocarcinoma (BA). The aim of this study is to determine the frequency and timing of LOH at the *TS *locus. In addition, intratumoral *TS *mRNA expression levels were measured in BA patients in order to determine whether these *TS *polymorphisms were associated with TS levels.

## Methods

### Patients and Samples

One hundred patients (73 males and 27 females) with gastroesophageal reflux disease (GERD), intestinal metaplasia (IM), dysplasia or Barrett's-associated adenocarcinoma (BA) were included in this study. Written informed consent was obtained from each patient according to institutional regulations.

Biopsies were performed by endoscopy or surgically from lower esophagus (squamous epithelium 3 cm above the gastroesophageal junction) (37 samples), IM (29 samples), dysplasia (13 samples), and BA (44 samples) where available. Since 18 patients had two or more different pathological tissue types (e.g.: IM and BA) in their lesions, multiple biopsies were taken from these 18 patients, one sample being taken from each tissue type (Table 1).

**Table 1:** Patient characteristics

The number of patients	100
Male	73
**Female**	27
**Age (median)**	65
**Pathological type (in descending order of)**	
**Reflux esophagitis**	30
**Intestinal metaplasia**	22 (※)
**Dysplasia**	4
**Barrett's adenocarcinoma**	44 (※※)
**Race**	
**Caucasian**	89
**Asian**	6
**Hispanic**	3
**African-American**	1
**Native American**	1
**TS genotype in normal tissue**	
**2R/2R**	24
**2R/3R**	57
**3R/3R**	19

※ Including 4 patients from each of whom 2 samples of different diseased tissues were taken (e.g: reflux esophagitis and intestinal metaplasia).

※※ Including 9 patients from each of whom 2 samples of different diseased tissues were taken, and 5 patients from each of whom 3 were taken.

In each patient, normal squamous epithelium 20 cm below the incisors was taken by endoscopy as a control sample. This study has been approved by the ethics committee in the University of Southern California, and has been performed in accordance with the Declaration of Helsinki.

In 32 out of 44 BA patients, the data of *TS *genotype and LOH status were already reported previously [21].

### Microdissection

The frozen samples were embedded in optimal cutting temperature (O.C.T.) compound (Sakura Finetek U.S.A., Inc., Torrance, CA) and cut into serial sections with a thickness of 20 μm. Sections were mounted on uncoated glass slides and stored at -80°C. For histological diagnosis, one slide was stained with H&E and evaluated by a pathologist. Before microdissection, sections were air-dried, fixed in 70% ethanol for 3 minutes and washed in H_2_O for 2 min. Afterwards, they were stained with nuclear fast red (NFR, American MasterTech Scientific, Inc., Lodi, CA) for 20 seconds and again washed in H_2_O for 30 seconds. Samples were then dehydrated in a stepwise manner with 70% ethanol, 95% ethanol and 100% ethanol for 30 seconds each, followed by incubation in xylene for 5 minutes and complete air-drying. Normal squamous cell control samples were dissected from the slides using a scalpel. All other sections were selectively dissected by laser captured microdissection (P.A.L.M. microsystem, Leica, Wetzlar, Germany) according to the standard procedure [22]

### RNA Isolation and cDNA Synthesis

RNA isolation from OCT-embedded specimens was done according to a proprietary procedure of Response Genetics, Inc. (US patent number 6,248,535) [23]. In brief, tissue samples were placed in 4 M dithiothreitol (DTT)-GITC/sarc (4 M guanidinium isothiocyanate, 50 mM Tris-HCl, pH 7.5, 25 mM EDTA) (Invitrogen: cat. no. 15577-018). To the tissue suspensions were added 50 μL of 2 M sodium acetate, pH 4.0, followed by 600 μL of freshly prepared phenol/chloroform/isoamyl alcohol (250:50:1). The suspensions were centrifuged at 13,000 rpm for 8 min in a chilled (8°C) centrifuge. The upper aqueous phase was removed and combined with glycogen (10 μL) and 300–400 μL of isopropanol. The tubes were left standing at -20°C for 30–45 min to precipitate the RNA. After centrifugation at 13,000 rpm for 7 min in a chilled (8°C) centrifuge, the supernatant was carefully poured off and the pellet was re-suspended in 50 μL of 5 mM Tris. Afterwards, cDNA was prepared as previously described [24]. In brief, 20 μL 5× Moloney murine leukemia virus (MMLV) buffer (containing 250 mmol/L Tris-HCl [pH 8.3], 375 mmol/L KCl, and 15 mmol/L MgCl2; Life Technologies, Gaithersburg, Md.), 10 μL dithiothreitol (100 mmol/L; Life Technologies), 10 μL dNTP (each type 10 mmol/L; Amersham Pharmacia Biotech), 0.5 μL random hexamers (50 OD dissolved in 550 μL of 10 mmol/L Tris-HCl [pH 7.5], and 1 mmol/L EDTA; Amersham Pharmacia Biotech), 2.5 μL bovine serum albumin (3 mg/ml in 10 mmol/L Tris-HCl [pH 7.5], Amersham Pharmacia Biotech), 2.5 μL RNAse inhibitor (5× 1000 units; Amersham Pharmacia Biotech), and 5 μL MMLV reverse transcriptase (200 U/μL; Life Technologies), added to a total volume of 50.5 μL. Reaction temperatures were first at 26°C for 8 min, then at 42°C for 45 min,, finally at 95°C for 5 min.

### Real-time reverse transcriptase polymerase chain reaction (RT-PCR) quantification of mRNA expression

Quantitation of *TS *mRNA and an internal reference gene (β-actin) was done using a fluorescence-based real-time detection method (ABI PRISM 7900 Sequence Detection System (TaqMan^®^) Perkin-Elmer (PE) Applied Biosystem, Foster City, CA, USA), as described previously [24-26]. The PCR reaction mixture consisted of each primer (1200 nM), 200 nM probe, 0.4 U of AmpliTaq Gold Polymerase, 200 nM concentrations of each of dATP, dCTP, dGTP and dTTP, 3.5 mM MgCl2 and 1× Taqman Buffer A containing a reference dye, to a final volume of 20 μl (all reagents from PE Applied Biosystems, Foster City, CA, USA). Cycling conditions were 50°C for 2 min, 95°C for 10 min, followed by 46 cycles at 95°C for 15 s and 60°C for 1 min. The sequence of primers and probes of *TS *and *β-actin *was described previously [13].

Gene expression values (relative mRNA levels) are expressed as ratios (differences between the Ct values) between the genes of interest (TS in this case) and an internal reference gene (β-actin) that provides a normalization factor for the amount of RNA isolated from a specimen.

### DNA Extraction, genotyping

**Figure 1:**
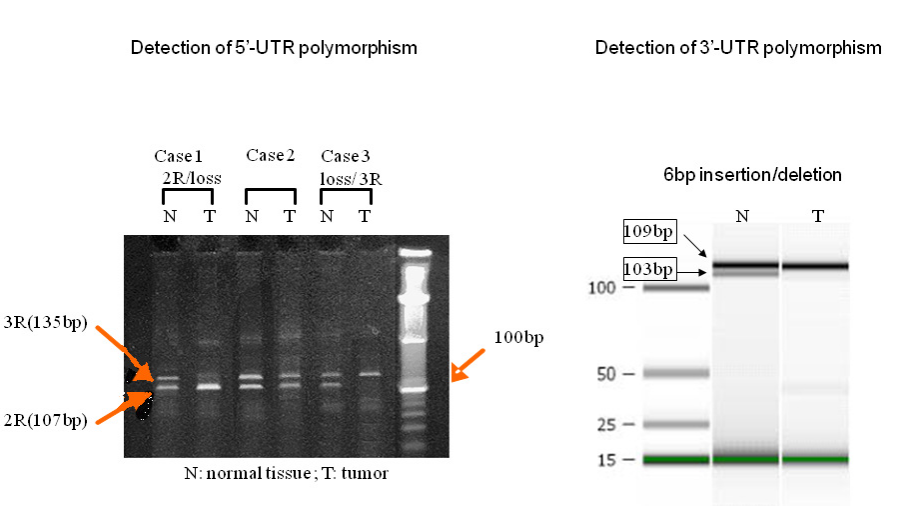
**A (left side): Detection of TS 5'-UTR polymorphism**. Thymidylate synthase (TS) 5'-UTR genotype analysis in matched normal (*N*) and tumor (*T*) DNA. The upper and lower bands represent PCR products from amplification of the TS segment containing 3R and 2R, respectively. Each patient has a heterozygous 2R/3R genotype in normal tissue, as indicated by the presence of both bands. Case 1: Loss of heterozygosity (LOH) gives rise to a tumor with a 2R/loss genotype. Case 2: LOH does not occur. Case 3: LOH gives rise to a tumor with a 3R/loss genotype. **1B (right side): Detection of TS 3'-UTR polymorphism**. Thymidylate synthase (TS) 3'-UTR genotype analysis in matched normal(*N*) and tumor (*T*) DNA. The middle lane shows the double bands in 109 bp and 103 bp, representing the heterozygous 6 bp insertion/deletion. The right lane shows the single band in 109 bp, representing the loss of the 103 bp band.

Genomic DNA was extracted using the QIAamp kit (Qiagen, Valencia, CA). The promoter region (5'-UTR) of the *TS *gene was amplified by the polymerase chain reaction using the primers described previously [13]. The PCR primers are designed to flank the region of the tandem repeats. PCR was performed using the conditions described previously [7]. The PCR products were analyzed by electrophoresis on a 10% TBE-urea polyacrylamide gel (Invitrogen Corp., Carlsbad, CA). We obtained PCR fragments with an estimated length of 107 and 135 bp, which represent the two- and three-repeat (2R and 3R) sequences, respectively. The *TS *genotypes were classified into 2R-homozygote (2R/2R), 3R-homozygote (3R/3R), and 2R/3R heterozygote in normal tissue. LOH in 2R/3R heterozygous individuals was detected by comparing the genotype of the normal control sample obtained in the upper and lower esophagus where GERD, IM, dysplasia, or BA were present in the same patient. LOH was indicated by loss of either the 2R (3R/loss) or the 3R band (2R/loss) (Fig 1A). A G/C polymorphism in triple repeat sequence was analyzed as described previously [9].

3'-UTR polymorphism was determined using PCR. The primers were as follows: forward primer 5'-GCTGAGTAACACCATCGATCATG-3' and reverse primer 5'-GCGTGGACGAATGCAGAAC-3'. Cycling conditions were 50°C for 2 min, 95°C for 10 min, followed by 46 cycles at 95°C for 15 s and 64°C for 1 min. PCR products which contained the 6 bp polymorphic region were loaded in an Agilent 2100 bioanalyzer (Agilent, Palo Alto, CA), and the 6 bp difference was detected (Fig. 1B).

### Statistical analysis

*TS *mRNA expression levels in esophageal adenocarcinoma were compared between the *TS *genotype groups by using the Mann-Whitney U test for comparing two groups, and the Kruskal-Wallis test for 3 groups. All reported P values are two-sided and statistical significance was set at a P-value of less than 0.05.

## Results

### Frequency of LOH at *TS *5'-UTR locus in IM-BA sequence

The patients with informative heterozygous genotype in the 5'-UTR polymorphism in their normal squamous tissues were evaluated the presence of LOH in their IM, dysplasia, and BA tissues. The frequencies of LOH in each pathologic group, which were calculated on the basis of the number of samples examined are shown in Table 2. Although no samples from GERD patients with LOH at the *TS *locus were observed in the lower esophageal mucosa (0/22 samples)), 6 out of 21 samples (28.6%) had LOH in IM, 2 out of 7 (28.6%) in dysplasia, and 10 out of 25 (40.0%) in BA.

**Table 2:** Frequency of 5'-UTR loss of heterozygosity (LOH) in low esophagus, IM, dysplasia and BA tissues

Low esophagus (LE) (n = 37)					
Genotype in normal tissues	2R/2R	2R/3R			3R/3R

Number of samples	8	22			7

Genotype in LE tissues		2R/loss	2R/3R	3R/loss	
Number of samples		0	22	0	
Frequency		0%	100%	0%	

					
**Intestinal metaplasia (IM) (n = 29)**					

Genotype in normal tissues	2R/2R	2R/3R			3R/3R

Number of samples	3	21			5

Genotype in IM tissues		2R/loss	2R/3R	3R/loss	
Number of samples		5	15	1	
Frequency		23.80%	71.40%	4.80%	

					
**Dysplasia (n = 13)**					

Genotype in normal tissues	2R/2R	2R/3R			3R/3R

Number of samples	4	7			2

Genotype in dysplasia tissues		2R/loss	2R/3R	3R/loss	
Number of samples		1	5	1	
Frequency		14.30%	71.40%	14.30%	

					
**Barrett associated adenocarcinoma (BA) (n = 44)**					

Genotype in normal tissues	2R/2R	2R/3R			3R/3R

Number of samples	15	25			4

Genotype in BA tissues		2R/loss	2R/3R	3R/loss	
Number of samples		3	15	7	
Frequency		12%	60%	28%	

The frequency of LOH at the 3'-UTR locus was also evaluated in cancer patients. In 14 informative heterozygous patients, the frequency of LOH was 35.7%, which was close to the frequency of LOH at the 5'-UTR locus. There were 3 patients who had LOH at the 5'-UTR locus and also had a heterozygous genotype at the 3'-UTR locus, and all 3 patients additionally showed LOH at the 3'-UTR locus.

### *TS *5'-UTR genotype in different histological tissues from the same patient

Eleven BA patients had IM and/or dysplasia adjacent to BA, and 9 out of 11 patients showed the heterozygous 2R/3R genotype in their 5'-UTR loci (Table 3). Of these 9 patients, 4 had LOH in their BA tissues. Of these 4, 2 also showed LOH in their precursor tissues, such as IM or dysplasia, whereas the other 2 patients showed LOH only in their BA tissues, not in the precursor tissues. This data suggests that LOH sometimes occurs in the early stages of carcinogenesis, and that the timing of the appearance of LOH is not always the same in every patient.

**Table 3:** *TS *genotype of Barrett's-associated adenocarcinoma patients who had adjacent intestinal metaplasia and/or dysplasia

Pt. No.	Normal	Metaplasia	Dysplasia	Adenocarcinoma
1	2R/3R	2R/3R	N/A	2R/3R
2	2R/3R	2R/loss	2R/loss	2R/loss
3	2R/2R	2R/2R	2R/2R	2R/2R
4	2R/3R	2R/3R	2R/3R	2R/3R
5	2R/3R	2R/3R	N/A	2R/3R
6	2R/3R	2R/3R	2R/3R	2R/3R
7	2R/3R	2R/3R	N/A	2R/loss
8	2R/3R	N/A	2R/3R	2R/3R
9	2R/2R	N/A	2R	2R/2R
10	2R/3R	N/A	2R/3R	loss/3R
11	2R/3R	N/A	loss/3R	loss/3R

### *TS *mRNA expression in BA tissue in 5'-UTR and 3'-UTR genotypes

**Figure 2:**
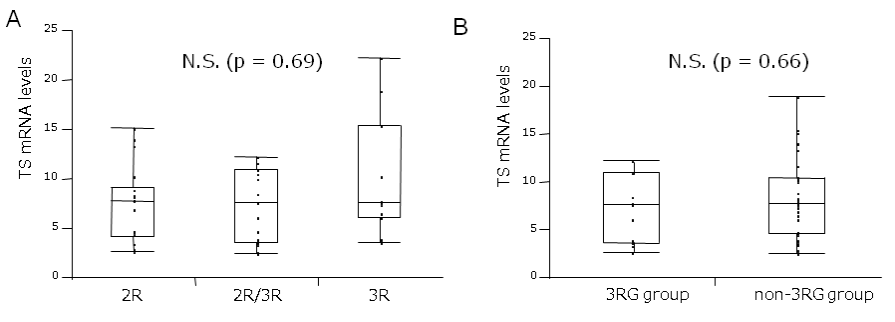
**Comparison of intratumoral *TS *mRNA levels with the 5'-UTR genotype**. A: Grouped by the number of repeats. No difference was observed between 2R/2R, 2R/3R, and 3R/3R groups. B: Grouped by the number of repeats and G/C SNPs. The patients with 2R/3RG, 3RC/3RG, and 3RG/3RG genotypes were classified as the 3RG group, while those with 2R/2R, 2R/3RC, and 3RC/3RC were classified as the non-3RG group. There was no difference in TS mRNA levels between these two groups. Boxes indicate the first and third quartiles (median inside); bars represent the range of values falling within 1.5-fold the interquartile range.

The comparison of *TS *mRNA expression levels in BA tissue in 5'-UTR genotypes are shown in Figs. 2A, B. When patients were classified as 2R group patients (2R/2R, 2R/loss), 3R group (3R/3R, loss/3R), and 2R/3R group, according to the number of repeats, no difference of median *TS *mRNA levels was observed between the groups (p = 0.69). When patients were classified in the 3RG group, the genotype that contained the 3RG allele, (2R/3RG, 3RC/3RG, 3RG/3RG) and the non-3RG group (2R/2R, 2R/3R, 3RC/3RC) according to the number of repeats and the G/C SNP, there was also no significant difference in median *TS *mRNA levels between these 2 groups (p = 0.66). There was also no difference in median TS mRNA levels between the patients with LOH and those without LOH in 5'UTR genotypes in 31 informative BA patients (p = 0.53).

**Figure 3:**
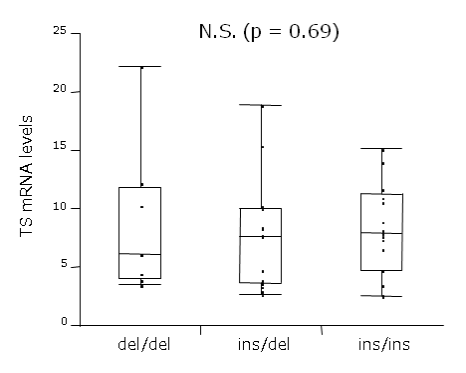
**Comparison of intratumoral *TS *mRNA levels with *TS *3'-UTR genotype**. No significant difference in TS mRNA levels was observed between the patients with ins/ins, ins/del, and del/del. Ins, 6 bp inserted allele; del, 6 bp deleted allele.

The same result was observed in 3'-UTR polymorphism, so that no significant difference was seen between ins/ins, ins/del, and del/del groups (p = 0.68) (Fig. 3).

## Discussion

LOH in tumor suppressor gene loci, such as 17p (*p53*), 18q (*DCC*), 9p (*CDKN2/p16*), 5q (*APC*), and 10q (*PTEN*), has been reported to be a common genetic alteration in the metaplasia-dysplasia-carcinoma sequence in Barrett's esophagus [20,27-33]. Several previous studies demonstrated that LOH in Barrett's esophagus was observed in the early stage of this sequence [20,28,29]. Suspiro *et al*. reported that LOH on 9p and 17p were detected in 35% and 39% of Barrett's esophagus patients without any evidence of adenocarcinoma, and concluded that the presence of these LOH may be useful markers for risk stratification within endoscopic surveillance [27].

In this study, we observed 28.6% of LOH at the *TS *5'-UTR locus even in IM tissue, which indicates that this genetic alteration may occur at some early stage of carcinogenesis. However, no LOH was observed in the lower esophageal tissues of GERD patients, suggesting that merely inflammation in the squamous epithelium is not a change sufficient to cause this genetic alteration, but that the replacement of the squamous epithelium by metaplastic columnar cells is necessary. Our data showed that, of 4 patients who had LOH in cancer tissue and whose precursor tissues were assessable, 2 had LOH in both cancer and precursor tissues, while 2 had LOH in cancer tissue only, but not in the precursor tissues. These data indicate that the timing of this genetic alteration may vary between individuals.

In our 11 BA patients who had IM and/or dysplasia adjacent to cancer, the allelic loss pattern showed that the same allele copy was deleted in each individual patient between cancerous tissue and its precursor tissues. This observation suggests that BA has a monoclonal tumor cell population through its carcinogenesis step from IM to BA, and further confirms that IM and dysplasia are clonal precursors of BA. Zhuang *et al*. evaluated genetic clonality by using a polymorphic marker flanking the APC gene locus in 12 patients of BA as well as their IM and dysplasia, and found that identical alterations were observed in BA, dysplasia and some IM tissue [34]. Similar results have also been reported in the previous literature [35,36].

The reason why LOH occurs at the *TS *locus is still unclear. *TS *itself is probably not a target gene of LOH because it is an essential gene for DNA synthesis. If some unknown tumor suppressor genes are located close to the *TS *locus, the *TS *gene may be contained in the deleted DNA segment. Tran *et al*. examined the prevalence of LOH at 18p11 using six PCR-based polymorphic markers in non-small cell lung cancer, breast cancer, and glioblastoma, and found two frequency peaks at 18p11, suggesting that two potential tumor suppressor genes are present in chromosome 18p [37]. One of these regions is estimated to be located in between the two markers DS18S59 and D18S476, where the whole *TS *gene is located. The result of this study may support our hypothesis that unknown tumor suppressor genes are located close to the *TS *locus.

In our data, no relationship was observed between *TS *polymorphisms, either 5'-UTR or 3'-UTR, and *TS *mRNA expression. Whether *TS *polymorphisms in 5'-UTR or 3'-UTR are associated with *TS *mRNA expression levels has been a controversial topic for some years. Mandola *et al*. Reported, on the basis of in vitro data that this 5'-UTR polymorphism is related to the transcriptional activity of the *TS *gene [10]. In an vivo study, Morganti *et al*. found an association between *TS *mRNA expression and 5'-UTR polymorphism [38]. In contrast, Kawakami *et al*. quantified the *TS *mRNA level and the *TS *protein level, and found no relation between the genotype and the mRNA level, although the protein level was significantly related [7]. With respect to the 3'-UTR genotype, Mandola *et al. *reported that 6 bp deletion constructs had significantly decreased mRNA stability compared with 6 bp insertion constructs *in vitro*, and that the patients with an ins/ins genotype had significantly higher TS mRNA levels compared with those with del/del genotype *in vivo *[12]. In contrast, another investigation rshowed no association between 3'-UTR polymorphisms and TS mRNA levels [39]. Our previous data have shown neither 5'-UTR polymorphism nor 3'-UTR polymorphism to be associated with *TS *mRNA expression levels in esophageal adenocarcinoma [21], a finding that is supported by the present study.

## Conclusion

LOH at the *TS *locus may occur as an early event in the IM-BA sequence. Our next question is whether patients with LOH at the *TS *locus in their precursor tissue have a higher cancer risk than those who do not have LOH. For Barrett's esophagus patients, a prospective study with long-term surveillance is clearly needed.

## Competing interests

KDD is CEO of Response Genetics Inc. and owns stocks in Response Genetics Inc. PVD owns stocks in Response Genetics Inc.

## Authors' contributions

HK carried out the molecular genetic studies, participated in the design of the study, engaged in primer design, statistical analysis, and drafted the manuscript. KU carried out the molecular genetic studies and participated in the design of the study. JHP participated in the design of the study. DS, DV, and SS contributed in sample collection and carried out the molecular studies. KDD and PVD coordinated the study and helped to draft the manuscript.

## Pre-publication history

The pre-publication history for this paper can be accessed here:

http://www.biomedcentral.com/1471-2407/9/157/prepub
